# Activity Pattern of Urban Adult Students in an Eastern Mediterranean Society

**DOI:** 10.3390/ijerph13100960

**Published:** 2016-09-28

**Authors:** Issam Odeh, Tareq Hussein

**Affiliations:** 1 Department of Basic Sciences, Al Zaytoonah University of Jordan, P.O. Box 130, Amman 11733, Jordan; odehi.m@zuj.edu.jo; 2 Department of Physics, The University of Jordan, Amman 11942, Jordan; 3 Division of Atmospheric Sciences, Department of Physics, University of Helsinki, P.O. Box 48, FI-00014 UHEL, Helsinki, Finland

**Keywords:** time-spent, indoors, outdoors, traffic, exposure

## Abstract

Knowledge of human activity patterns is needed in air pollution exposure and health risk assessment. However, human activity patterns have never been evaluated in the Eastern Mediterranean societies. Therefore, we investigated the activity pattern of 285 subjects (17–63 years) in Amman, Jordan during October to November, 2015. The subjects spent >80% of their time indoors during weekend days and >85% on workdays. They spent ~4.8% and ~5.7% in transportation during weekend days and workdays, respectively. Males had a different activity pattern than females on weekend days, but both genders had similar activity patterns on workdays. On workdays, males spent less time indoors than females. The activity pattern found in this study is a bit different than that for North Americans and Europeans, who spend more time indoors and in transit. The activity pattern found in this study was very different than that observed for Koreans, who spent about 59% and 67% indoors on workdays and weekend, respectively. The main outcomes of this survey can be utilized in human exposure studies. This study and the upcoming future studies have been encouraged and supported by the regional WHO office in Amman.

## 1. Introduction

In addition to an air quality data-base, knowledge of human activity patterns is necessary in exposure assessment [1]. “Activity pattern” includes information about the activities conducted, as well as time spent in different environments. In addition to understanding and assessing the health effects of air pollution, physical activity (sleeping, sitting, walking, running, exercising, etc.) has also been found to be an important parameter in understanding the mental health of people [2,3,4].

Since the 1970s, this topic has been given great attention [5,6]. The United States and Canada were the first to report human activity patterns [7,8,9,10,11,12,13]. Due to their importance in investigations of exposure and health effects, human activity pattern data-bases were made available in the National Human Activity Pattern Survey (NHAPS) of 9386 interviews conducted between 1992 and 1994 in the United States [11,14], and the Canadian Human Activity Pattern Survey (CHAPS) of 2381 interviews conducted between 1996 and 1997 in four major Canadian cities [9].

In Europe, Schweizer et al. [15] reported the workday activity patterns of an adult urban population (1427 individuals, age 19–60 years) in seven European cities: Helsinki, Athens, Basel, Grenoble, Milan, Prague, and Oxford. A more extensive survey included 12,000 persons to report their time spent indoors with associated activity patterns in 5530 randomly-selected apartments and houses in Germany [16]. Dörre [17] reported the activity patterns of toddlers (*N* = 52, aged 2–3 years) in a nursery school and another two groups of medical students (*N* = 79 and 54, respectively). Hussein et al. [18] reported the activity pattern of 167 persons (age range: 2–93 years) during three time periods in Helsinki.

In other parts of the world, investigations of human activity patterns have been very rare. For example, Yang et al. [19] considered 31,634 individuals older than 10 years to report Koreans’ activity patterns in a survey during two consecutive days (a workday and a weekend day). Jim and Chen [20] analyzed how urban residents (598 valid questionnaires) spent their leisure time on workdays and weekends. In fact, Europeans and North Americans have similar activity patterns. It was also shown that Koreans spend less time indoors, and thus, their activity pattern is different than that in Western countries [19]. This indicates that cultural differences have a clear influence on human activity patterns.

However, activity patterns have never been investigated in the Eastern Mediterranean societies. Therefore, we investigated the activity pattern of 285 subjects (age range: 17–63 years) living in the Greater Municipality of Amman, Jordan during autumn 2015. The main outcomes of this survey can be utilized in human exposure studies to be conducted in the future. This study and the upcoming ones have been encouraged and supported by the regional WHO office in Amman, Jordan.

## 2. Materials and Methods

### 2.1. Design of the Questionnaire

We used a questionnaire which was previously designed by Hussein et al. [18] (but slightly modified and translated to Arabic) to report the time spent and environment of a selected group of university students and some members of their families. The reporting was at half-hourly time resolution during nine days, including five workdays (Sunday–Thursday) and two weekend days (Friday and Saturday). The questionnaire (in Arabic) included three main categories for the environment: indoor, outdoor, and transportation. The indoor category included five subcategories: home, university/school, work, shops, and others. The outdoor category included four subcategories: work, city, nature, and others. The city subcategory represents residential areas, city centers, etc. The nature subcategory represents national parks located within the city itself or forest areas near the city.

The questionnaire was designed on a single page with a table that included the environments in the left column. The residence time was reported by the individuals on a daily basis. It also included personal information: age, gender, profession, and place of residence. However, some participants did not provide information about their profession or their environment.

The paper questionnaire was converted into digital format for further processing and quality assurance check. The quality assurance check was mainly performed for the correctness of the personal information given by the subjects and the sum of their daily residence time as equaling 24 h per day. We excluded questionnaires that did not match 24 h per day. We also excluded questionnaires that had missing personal information (such as gender, age, etc.).

### 2.2. Individual Subjects

**Figure 1:**
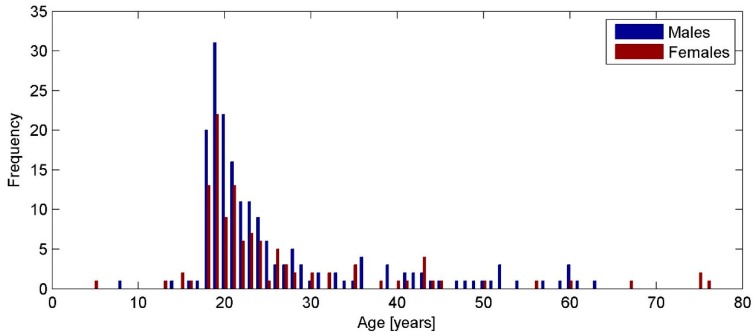
Distribution of subjects’ age according to the valid questionnaires.

The subjects reported their activities during October to November, 2015 (i.e., late autumn). More than 400 subjects participated in the survey. However, only 297 questionnaires (age range 5–76 years) were valid. Furthermore, we limited our analysis to a group of subjects in the age range 17–63 years; i.e., adults younger than the retirement age (63 years), because the number of subjects was small (about 4% of all subjects, 12 subjects) outside that age range. In total, we had 285 valid questionnaires included in the investigation, covering the age range 17–63 years (Figure 1). We had 197 subjects (121 males and 76 females) in the age range 17–24 years, which included the majority of the subjects (69%). The rest of the subjects (88, about 31%) were 58 males and 30 females in the age range 25–63 years.

## 3. Results

Regardless of age and gender, the subjects spent more or less about 80.7% of their time indoors (about 15 h at home and about 4 h in other indoor environments) on weekend days (Table 1). Furthermore, they spent ~14.5% of their time outdoors, and ~4.8% in transportation. With respect to workdays, the percentage of time spent indoors was about 85%, which was higher than that on weekend days. They also spent a longer time (~5.7%) in transportation on workdays than on weekend days. Consequently, the time spent outdoors on workdays was less than that on weekend days. It is also clear that shopping likely happened during the weekend; the time spent shopping on weekends was twice that spent on workdays. Similarly, more time was spent at home during the weekend than on workdays. Consequently, the time spent either at school or at work was greater on workdays than weekends, which is a trivial result. The time spent in traffic was rather the same for all days of the week. The time spent outdoors on workdays was more than that on weekends.

Looking more closely at the survey results with respect to gender, we found that males have a completely different activity pattern than females on weekend days (Table 1). Males spent about 78% indoors, 16% outdoors, and 6% in transportation on weekend days, whereas females spent about 84% indoors, 12% outdoors, and 4% in transportation. Gender is, in fact, one of the major factors affecting the activity pattern. For example, in our previous investigation for the activity pattern of people in Helsinki, we found out that teenage females spent 40% less time in outdoor environments than males [18].

Table 1 illustrates the time spent in different indoor environments compared to time spent outdoors and in transportation. It is clear that females spent more time at home than males. Females spent about 70% and 53% at home on weekends and workdays, respectively. Males spent about 60% and 46%, respectively. It looks like males spent more time (about 17%) at work during workdays than females (about 12%).

## 4. Discussion

The activity pattern is valuable information used in exposure studies. This kind of information has never been investigated in the Eastern Mediterranean countries. Therefore, we performed a survey of the activity pattern of 400 subjects living in the Greater Municipality of Amman, Jordan during late autumn of the year 2015. However, the analysis was limited to 285 subjects (age range 17–63 years), who continuously completed their questionnaire carefully. The majority of the subjects were younger than 25 years old, which means that our investigation was focused on adults in their early age. Therefore, we cannot make definite statements on the activity pattern changes with respect to age, which is a major factor in defining the type/style of the activity pattern.

As illustrated in the results section, the subjects spent about 80% of their time indoors on weekend days and about 85% on workdays. They also spent a longer time in transportation on workdays than on weekend days. Consequently, the time spent outdoors on workdays was less than that on weekend days. Compared to other communities, the activity pattern found in this study is a bit different than that for North Americans and Europeans [7,8,11,16]. For instance, Jenkins et al. [7] showed that the activity pattern of Californians was 87% indoors, 7% in enclosed transit, and 6% outdoors. However, the activity pattern found in this study was different than that observed for Koreans [19], who spent about 59% and 67% indoors on workdays and weekend days, respectively.

Previous studies anticipated that the differences in the time spent might occur individually, and these can be explained by gender, age, social status, season, etc. Schweizer et al. [15] concluded that more than 90% of the variance in the time spent indoors originated from differences between and within subjects, rather than between cities. Therefore, European and North American city residents have similar time spent patterns indoors and outdoors because they share rather similar life styles.

Age is expected to be another major factor in defining the type/style of activity patterns. Adults are expected to spend more time in traffic than youth and elderly individuals, whereas elderly individuals are expected to spend less time outdoors than the other age groups. In Helsinki, for example, subjects older than 50 years spent increasingly more time indoors as they age [18]. On the other hand, subjects younger than 50 years spent more time at home and less time at school as they age.

In general, ambient conditions have a clear effect on the activity pattern. For example, Hussein et al. [18] showed that during warm periods, the residence time indoors decreased and residence time outdoors increased, but the time spent in traffic was rather similar. McCurdy and Graham [10] analyzed the Consolidated Human Activity Database (CHAD [11]) and showed that season/temperature and day-type (workdays versus weekends) were also important for explaining time spent indoors by Americans. Leech et al. [8] also reported that Canadians spent less time outdoors in winter and less time indoors in summer. While this study was conducted during late autumn, we expect the activity pattern to be a bit different during the winter or summer seasons. However, a straight-forward expectation of the differences in the activity pattern between different seasons is rather uncertain, because Jordanians’ activity strongly depends on temperature and social events—this requires further investigation on a long-term basis.

It worth mentioning that this study in its current form has some limitations. For example, we did not have information about the family relationship between the subjects. This point is very important and needs to be considered in future investigations. It is also important to re-apply this survey by including a larger number of subjects with different professions and backgrounds. For example, we aimed at random sampling by asking the students and their family members to participate regardless of their profession, age, etc. However, the majority of subjects were the students themselves. In that sense, we ended up with a convenience sampling instead of random sampling.

## 5. Conclusions

The subjects spent more than 80% and more than 85% of their time indoors during weekend days and workdays, respectively. They also spent about 4.8% and 5.7% of their time in transportation during weekend days and workdays, respectively. This indicates that they spent less time outdoors during workdays than weekend days. Males had a different activity pattern than females on weekend days, but both genders had similar activity patterns on workdays. Males spent less time indoors than females, and more time outdoors than females.

The activity pattern found in this study is slightly different than that for North Americans and Europeans, who spend more time indoors and in transit, as shown in the discussion of the previous section. It is also very different than that observed for Koreans, who spent about 59% and 67% indoors on workdays and weekends, respectively.

The main outcomes of this survey can be utilized in human exposure studies [21]. This study and the upcoming ones have been encouraged and supported by the regional WHO office in Amman.

## Figures and Tables

**Table 1:** Residence time (hours: average ± std) with respect to gender.

							Indoor	Outdoor	Traffic
		Home	School	Work	Shop	Other	All		
Males	Weekends	14.3 ± 5.5	0.3 ± 1.7	2.1 ± 3.8	1.0 ± 1.5	1.1 ± 1.9	18.8 ± 4.5	3.9 ± 3.8	1.3 ± 2.2
	Workdays	11.0 ± 3.8	3.9 ± 3.9	4.1 ± 4.3	0.5 ± 1.2	0.6 ± 1.4	20.2 ± 3.7	2.4 ± 3.1	1.4 ± 1.8
Females	Weekends	16.8 ± 5.7	0.3 ± 1.8	1.1 ± 2.9	1.1 ± 1.6	1.0 ± 2.0	20.3 ± 3.9	2.9 ± 3.4	0.8 ± 1.1
	Workdays	12.8 ± 4.6	3.5 ± 3.5	2.8 ± 4.0	0.5 ± 1.2	0.9 ± 2.1	20.6 ± 3.1	2.0 ± 2.7	1.4 ± 1.2
Both	Weekends	15.3 ± 5.7	0.3 ± 1.7	1.7 ± 3.5	1.0 ± 1.5	1.1 ± 2.0	19.3 ± 4.3	3.5 ± 3.7	1.2 ± 1.9
	Workdays	11.7 ± 4.2	3.8 ± 3.8	3.6 ± 4.2	0.5 ± 1.2	0.8 ± 1.7	20.4 ± 3.5	2.3 ± 3.0	1.3 ± 1.6
